# Population-level median cycle threshold (Ct) values for asymptomatic COVID-19 cases can predict the trajectory of future cases

**DOI:** 10.1371/journal.pone.0281899

**Published:** 2023-03-09

**Authors:** Naila Shoaib, Asim Iqbal, Farhad Ali Shah, Wajeeha Zainab, Maham Qasim, Noore Zerqoon, Muhammad Omer Naseem, Rimsha Munir, Nousheen Zaidi

**Affiliations:** 1 Cancer Biology Lab, Institute of Microbiology and Molecular Genetics, University of the Punjab, Lahore, Pakistan; 2 Cancer Research Centre (CRC), University of the Punjab, Lahore, Pakistan; 3 Hormone Lab, Lahore, Pakistan; 4 Institute of Learning Emergency Medicine, University of Health Sciences, Lahore, Pakistan; Imam Abdulrahman Bin Faisal University, SAUDI ARABIA

## Abstract

**Background:**

Recent studies indicate that the population-level SARS-CoV-2 cycle threshold (Ct) values can inform the trajectory of the pandemic. The presented study investigates the potential of Ct values in predicting the future of COVID-19 cases. We also determined whether the presence of symptoms could change the correlation between Ct values and future cases.

**Methods:**

We examined the individuals (n = 8660) that consulted different sample collection points of a private diagnostic center in Pakistan for COVID-19 testing between June 2020 and December 2021. The medical assistant collected clinical and demographic information. The nasopharyngeal swab specimens were taken from the study participants and real-time reverse transcriptase polymerase chain reaction (RT-PCR) was used to detect SARS-CoV-2 in these samples.

**Results:**

We observed that median Ct values display significant temporal variations, which show an inverse relationship with future cases. The monthly overall median Ct values negatively correlated with the number of cases occurring one month after specimen collection (*r* = -0.588, *p* <0.05). When separately analyzed, Ct values for symptomatic cases displayed a weak negative correlation (r = -0.167, p<0.05), while Ct values from asymptomatic cases displayed a stronger negative correlation (r = -0.598, p<0.05) with the number of cases in the subsequent months. Predictive modeling using these Ct values closely forecasted the increase or decrease in the number of cases of the subsequent month.

**Conclusions:**

Decreasing population-level median Ct values for asymptomatic COVID-19 cases appear to be a leading indicator for predicting future COVID-19 cases.

## Introduction

Real-time polymerase chain reaction (RT-PCR) is the primary workhorse in molecular diagnostics and approved gold standard for the diagnosis of SARS-CoV-2 [1]. It has been extensively used for high-throughput screening and early diagnosis of COVID-19 and other viral infections. The cycle threshold (Ct) value is the number of polymerase chain reaction (PCR) cycle at which the fluorescence signal crosses the defined threshold. The higher the amount of viral RNA, the earlier the threshold is reached, giving a lower PCR cycle number (Ct value). Hence, the Ct value is considered as a surrogate marker for the viral load and is reciprocally related to it. However, many factors affect the Ct values, including sample quality, type, and nucleic acid extraction method. Hence, Ct values are not directly comparable between assays [2, 3]. Many research groups, including ours, have reported that Ct values may have limitations in predicting COVID-19 severity and thus must be used with caution [4].

Although there are challenges to relying on single Ct values for individual-level decision-making, population-level Ct values are shown to predict the trajectory of the pandemic [5–7]. More recently, Phillips et al. have reported that the temporal trends in SARS-CoV-2 Ct values could predict future COVID-19 cases at an individual hospital level [8]. The presented work further explores the association between temporal trends in population-level Ct values and the number of future cases. We examined whether the median of population-level Ct values could be used to predict the trajectory of COVID-19. Moreover, we determined whether the distinction between symptomatic and asymptomatic patients would affect the prediction capability of the population-level Ct values.

## Methodology

### Study-population, ethics, and sample collection

This study includes patients (n  =  8660) sampled for COVID-19 testing at different collection points of a private diagnostic center (Hormone Lab) spaned across Punjab–the most populated province in Pakistan. These collection points are located in all the major cities of the Punjab, including Lahore, Gujranwala, Faisalabad, Multan, Gujrat, and Rawalpindi. The data was collected from June 2020 to December 2021. The institutional biosafety Committee-Hormone lab approved the study protocol for human subjects (Ethical Approval Number HM/ 56–06). Written informed consent was obtained from the participants or their guardians (in case of minors) before sample collection and data was fully anonymized before use. A confirmed case of COVID-19 was defined as having a positive result through real-time reverse-transcriptase-polymerase-chain-reaction (RT-PCR) assay of nasopharyngeal swab specimens. Only laboratory-confirmed cases were included in the analysis.

### RNA extraction and RT-PCR

Deep nasal cavity swab (nasopharyngeal) samples were collected from patients, and viral RNA was extracted by using “FavorPrep™ Viral Nucleic Acid Extraction Kit” and “Systaaq Diagnostic Products SuperExract 32 instrument” according to the manufacturer’s instructions. SARS‐CoV‐2 was detected by RT‐PCR assay using commercially available “*Sansure;* Novel Coronavirus (2019-nCoV) Nucleic Acid Diagnostic Kit” and “Systaaq Diagnostic Products AB QuantGene instrument” according to the manufacturer’s protocol. This kit include primers/probes that are specific for the open reading frame of 1ab (ORF1ab) gene (probe labeled 2 with FAM) and nucleocapsid protein (N) gene (probe labeled with VIC) of SARS-CoV-2. In addition, the kit also contain primers and a probe (labeled with CY5) for the human RNase P gene as an endogenous internal control for specimen integrity, nucleic acid isolation, amplification, and detection. This assay includes Ct values for the Orf1ab and N gene obtained from *Sansure Biotech kit*, and the Ct value <40 was used for this analysis.

### Statistical analysis

Statistical analyses were performed using Microsoft Excel and GraphPad Prism 9. One way ANOVA testing was used to compare monthly average Ct values. Positivity rate in each month is determined by the following formula:

% Positivity =

Correlations between Ct values from different patient populations and total cases were acquired using Pearson’s test. An alpha of 0.05 was used for all tests. A predictive model for future cases was created by doing a simple linear regression analysis on data from the Ct values.

## Results

### Ct values decrease with the surge of COVID-19 cases

**Fig 1:**
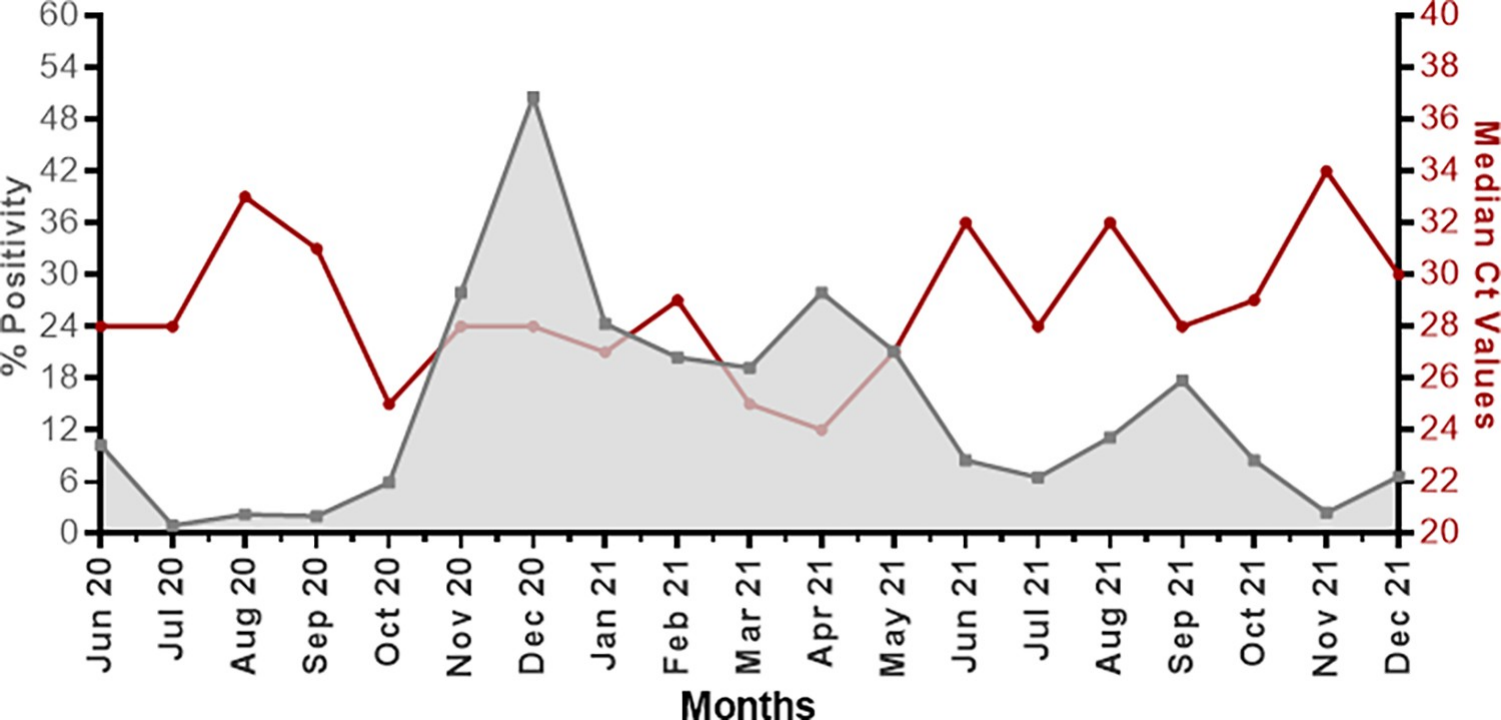
Temporal variations in COVID-19 percentage positivity and population-level median Ct values. The graph displays the COVID-19 percentage positivity per month (*left y-axis*) and monthly median Ct values (*right y-axis*) between June 2020 and December 2021.

For the present study, we tested 8660 participants for SARS-CoV 2, out of which 936 (10.8%) were COVID-19 positive. **Fig 1** displays the trends for monthly median Ct values and monthly percentage positivity for COVID-19 from June 2020 to December 2021. We observed that population-level Ct values change over time. Furthermore, an inverse pattern for the two variables was observed, i.e., as the rate of positive cases decreased, the median Ct values of positive cases reciprocally increased. We can also deduce that declining Ct values precede increasing SARS-CoV-2 positivity rate. Hence, population level Ct values could be a useful epidemiological early-warning indicator.

### Ct values correlate with future case numbers

**Fig 2:**
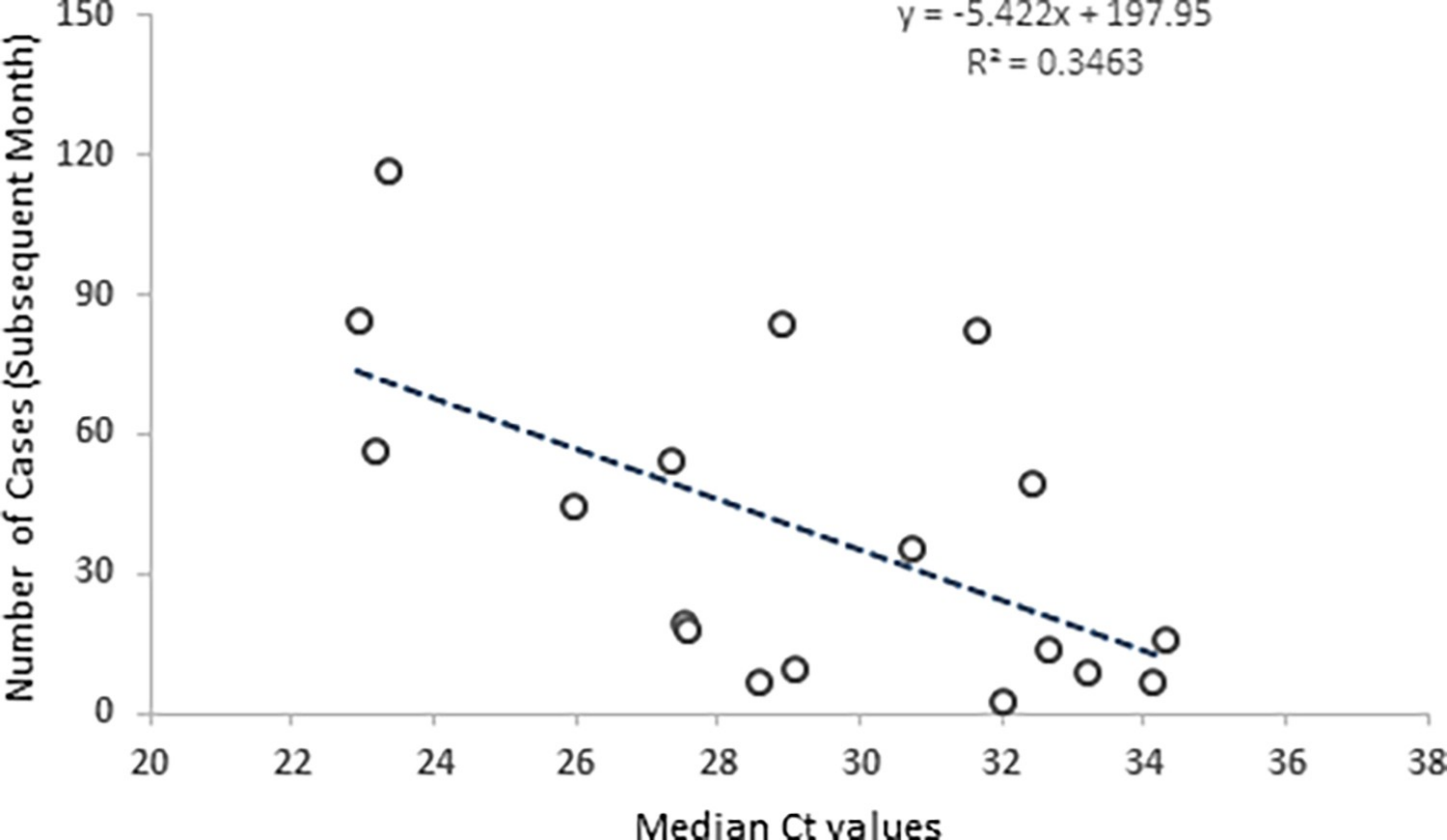
Correlation between Ct values and number of cases in the subsequent month. The correlation between monthly median Ct values and the number of positive cases in the subsequent months (r = -0.588, p<0.05).

To further assess whether Ct values could predict future caseloads, we performed a linear regression analysis between monthly median Ct values and the number of positive COVID-19 cases in the subsequent months. The monthly median Ct values negatively correlated with the number of cases occurring one month after specimen collection (*r* = -0.588, *p* <0.05) (**Fig 2**).

**Fig 3:**
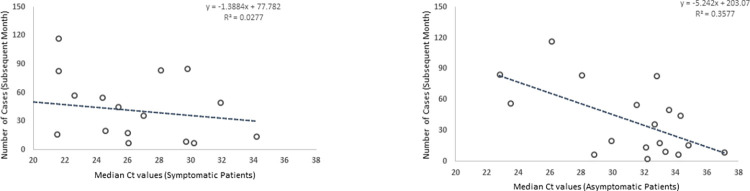
Correlation between Ct values from symptomatic and asymptomatic patients and the number of cases in the subsequent months. **(a)** Correlation between Ct values for symptomatic patients with the number of positive cases in the subsequent months (r = -0.167, p<0.05). **(b)** Correlation between Ct values for asymptomatic patients with the number of positive cases in the subsequent months (r = -0.598, p<0.05).

Next, we performed a regression analysis between monthly median Ct values of symptomatic or asymptomatic cases with the number of positive COVID-19 cases in the subsequent months (**Fig 3**). Ct values for symptomatic patients negatively correlated (r = -0.167, p<0.05) with the number of positive COVID-19 cases in the subsequent months (**Fig 3A**). Nonetheless, a comparatively stronger negative correlation (r = -0.598, p<0.05) was observed between Ct values from asymptomatic patients and the number of positive COVID-19 cases in the subsequent months (**Fig 3B**).

### Correlation between Ct values and future case numbers can be used to estimate future trends in caseloads

**Fig 4:**
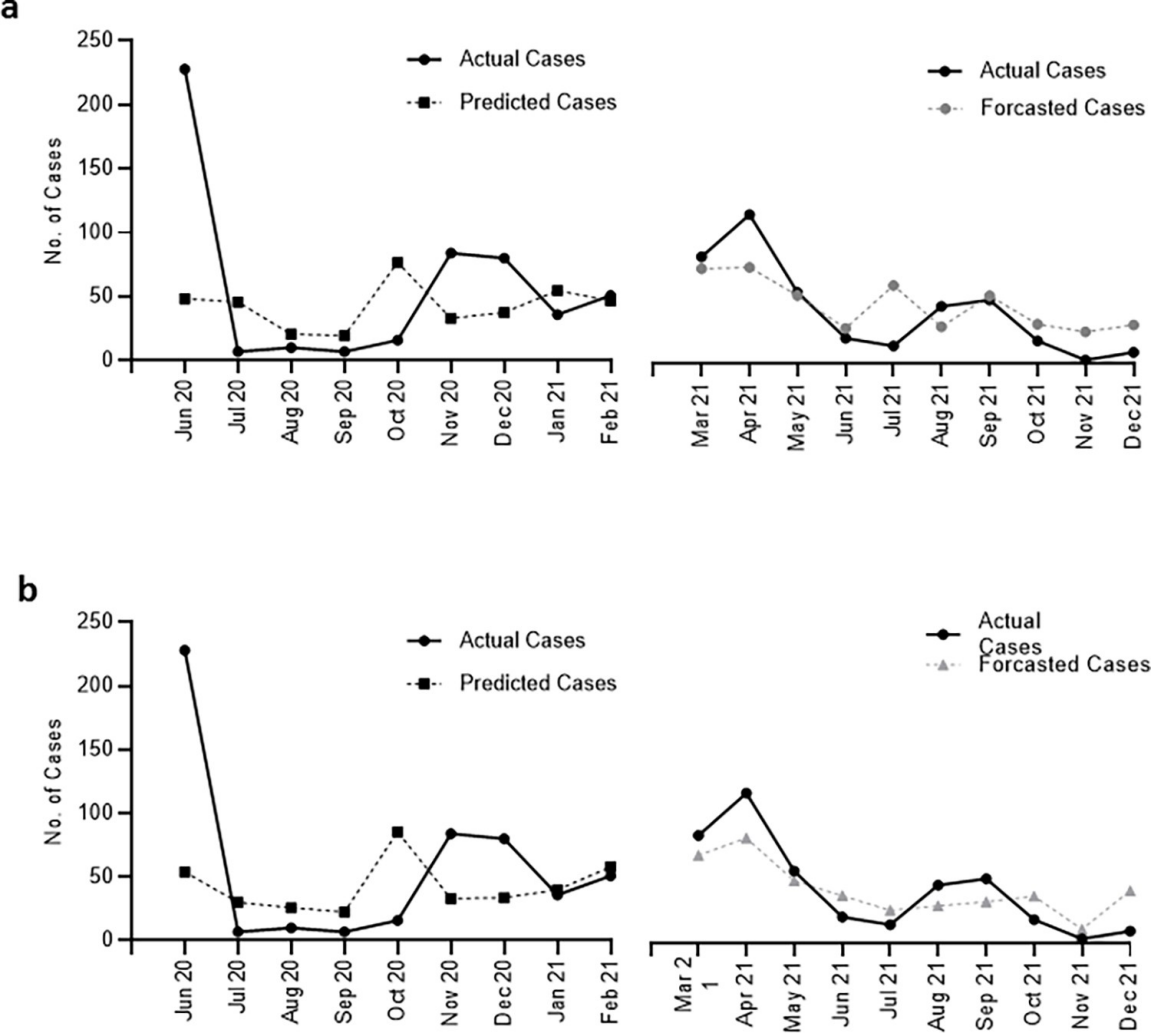
Actual and predicted cases using a linear regression model generated from monthly median Ct values. **(a)** The model was generated using Ct values for all COVID-19 cases. **(b)** The model was generated using Ct values of asymptomatic COVID-19 cases. The models were generated with Ct values of COVID-19 cases between Jun 2020 and Feb 2021. Data collected from Mar 2021 until Dec 2021 were used to validate the forecasting estimates.

Next, we sought to determine whether the correlation between Ct values and future cases could be leveraged to predict the trajectory of COVID-19 cases. We used linear regression models generated from the Ct values for all positive cases between Jun 2020 and Feb 2021 and the total number of Covid-19 positive cases in subsequent months. Data collected from Mar 2021 until Dec 2021 were used to validate the forecasting estimates (**Fig 4A**). The Pearson correlation between observed and forecasted data was 0.789 (**S1A Fig**), and the root mean square error (RMSE) was 23.5 cases.

As the correlation between Ct values from asymptomatic patients and total cases in the subsequent months was slightly strong, we used linear regression models generated between these variables for the data collected between Jun 2020 and Feb 2021. Data collected from Mar 2021 until Dec 2021 were used to validate the forecasting estimates (**Fig 4B**). The Pearson correlation between observed data and forecasted data was 0.879 (**S1B Fig**), and the root mean square error (RMSE) was 19.8 cases. Our data indicate that this method could closely predict whether cases would increase or decrease in the near future.

## Discussion

The current diagnostic methods for SARS-CoV-2 detection are generally of two types. The first type is molecular-based that detects nucleic acid presence, like real-time polymerase chain reaction (RT-PCR), multiplex RT-PCR [9, 10] and reverse-transcription loop-mediated isothermal amplification (RT-LAMP) [11]. The other type is immunological-based that detects antigens or antibodies in patient’s samples, like enzyme-linked immunosorbent assay (ELISA), lateral flow assay (LFA), chemiluminescent immunoassay (CLIA), and neutralization assay. However, immunological based assays give 0–30% false positive results [12]. Thus, PCR applications are much suitable in the diagnosis of SARS-CoV-2 infections.

Different clinical and demographical factors are associated with COVID-19 severity [13]. Previous studies have suggested that population-level Ct values could be used as an early warning indicator for the surge in COVID-19 cases. Our study focuses on the significance of population-level median Ct values in predicting the trajectory of the pandemic. We observed that median Ct values display significant temporal variations through the course of the pandemic. Temporal variations in the median Ct values and number of cases display an inverse relationship, i.e., as the number of cases decreased, the median Ct values reciprocally increased. More specifically, declining median Ct values preceded increasing SARS-CoV-2 positivity rate. Hence, we confirm the previous studies that population-level median Ct values could be helpful epidemiological early-warning indicators.

We observed a negative correlation between median Ct values and the number of cases in the subsequent months. It has been hypothesized that the primary factor driving this relationship is the temporal increase in individual Ct values over the course of an infection. During the pandemic, when there is a surge in COVID-19 cases, more individuals will be infected and thus Ct values decreased. When the number of cases decreases, more individuals will be farther along in their infections and thus Ct values increased.

Next, we sought to determine the impact of the presence of symptoms on correlations between Ct values and future cases. We observed that Ct values for the asymptomatic patients were most strongly associated with the total number of cases observed in the subsequent month, suggesting that low Ct values among asymptomatic individuals may be a more robust indicator for future surges in COVID-19 cases. One primary reason for this occurrence could be that asymptomatic patients represent a more random selection of individuals from the overall population; hence, their Ct values could better indicate the overall spread of the disease within the population. In contrast, symptomatic patients primarily represent a self-selected population that developed symptoms and underwent the COVID-19 test. Social behaviors are also possibly different between the asymptomatic and symptomatic groups. For instance, asymptomatic individuals would be more likely to engage in social activities and gatherings. These behavioral differences may also make the asymptomatic group a more significant driver of future surges in COVID-19 cases and, thus, a better indicator of measuring community spread [14]. Moreover, the viral dynamics significantly vary between asymptomatic and symptomatic individuals, which may also affect the correlation between the Ct values and future cases. For example, symptomatic patients can take considerably longer to clear the infection, increasing the proportion of late infections detected in the presented analysis [8].

Estimating the future surges in COVID-19 cases would provide better guidance for better resource allocation. The presented study shows that decreasing Ct values, particularly in the asymptomatic group, are the leading indicator for predicting future COVID-19 cases. Further studies are warranted to investigate the generalizability of these results.

## Supporting information

S1 Fig Pearson correlation between observed data and forecasted values. (a) Correlation between actual cases and cases predicted using the model generated with overall Ct values for all cases. (r = 0.789, p<0.05). (b) Correlation between actual cases and cases predicted using the model generated with Ct values for asymptomatic cases. (r = 0.879, p<0.05).
(PPTX)

S1 Table Number of SARS-CoV-2 positive individuals and the total tests performed in each month. (DOCX)

S2 Table 95% Confidence Interval of the monthly Ct values. (DOCX)

S1 Dataset Minimal data set for the current study. (XLSX)
