# Supplementary figures and images for: Population-level median cycle threshold (Ct) values for asymptomatic COVID-19 cases can predict the trajectory of future cases
Source: PLoS One. 2023 Mar 9;18(3):e0281899. doi: 10.1371/journal.pone.0281899 (PMC9997994; doi:10.1371/journal.pone.0281899)

## Slide 1
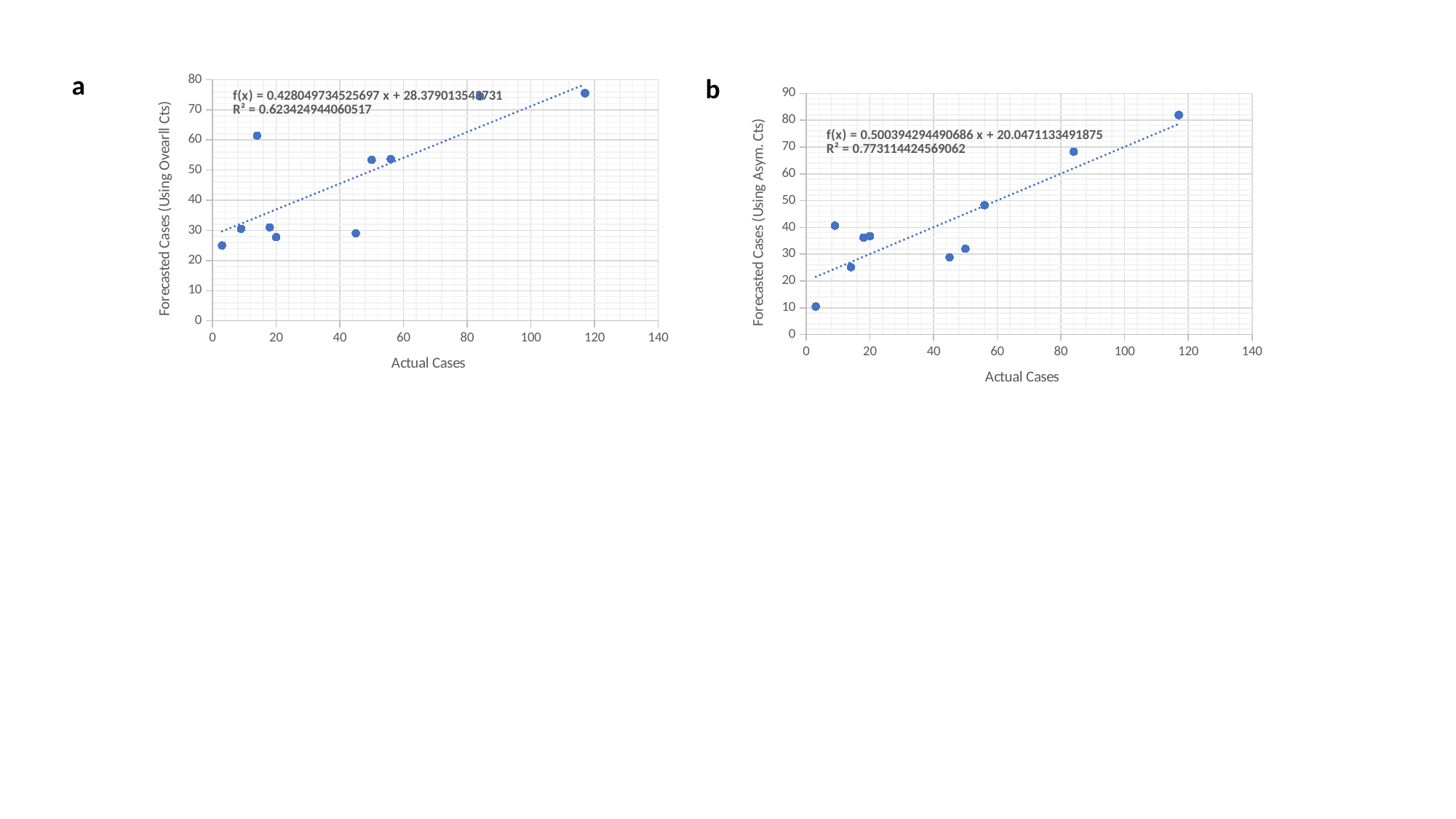

a
### Chart
| Category | |
|---|---|b
### Chart
| Category | |
|---|---|

Supplement: S1 Fig — (a) Correlation between actual cases and cases predicted using the model generated with overall Ct values for all cases. (r = 0.789, p<0.05). (b) Correlation between actual cases and cases predicted using the model generated with Ct values for asymptomatic cases. (r = 0.879, p<0.05). (PPTX) [file pone.0281899.s001.pptx]
