# Supplementary material for: Population-level median cycle threshold (Ct) values for asymptomatic COVID-19 cases can predict the trajectory of future cases
Source: PLoS One. 2023 Mar 9;18(3):e0281899. doi: 10.1371/journal.pone.0281899 (PMC9997994; doi:10.1371/journal.pone.0281899)
Supplement: S1 Table — (DOCX) [file pone.0281899.s002.docx]

| **Month** | **No. of positive individuals** | **Total no. of tests performed** |
| --- | --- | --- |
| **Jun-20** | 228 | 2237 |
| **Jul-20** | 7 | 739 |
| **Aug-20** | 10 | 447 |
| **Sep-20** | 7 | 353 |
| **Oct-20** | 16 | 273 |
| **Nov-20** | 85 | 305 |
| **Dec-20** | 83 | 164 |
| **Jan-21** | 36 | 148 |
| **Feb-21** | 55 | 270 |
| **Mar-21** | 84 | 437 |
| **Apr-21** | 117 | 420 |
| **May-21** | 57 | 270 |
| **Jun-21** | 20 | 234 |
| **Jul-21** | 14 | 216 |
| **Aug-21** | 45 | 406 |
| **Sep-21** | 50 | 283 |
| **Oct-21** | 18 | 212 |
| **Nov-21** | 3 | 124 |
| **Dec-21** | 9 | 136 |
