# Supplementary material for: Population-level median cycle threshold (Ct) values for asymptomatic COVID-19 cases can predict the trajectory of future cases
Source: PLoS One. 2023 Mar 9;18(3):e0281899. doi: 10.1371/journal.pone.0281899 (PMC9997994; doi:10.1371/journal.pone.0281899)
Supplement: S2 Table — (DOCX) [file pone.0281899.s003.docx]

| **Month** | **Ct value** | **95% Confidence Interval (CI)** |
| --- | --- | --- |
| June 2020 | 27.80696 | 27.066-28.547 |
| July 2020 | 27.51429 | 23.682-31.347 |
| August 2020 | 33.04 | 29.610-36.470 |
| September 2020 | 31.364 | 27.401-35.327 |
| October 2020 | 24.64286 | 21.638-27.648 |
| November 2020 | 28.37833 | 26.739-30.018 |
| December 2020 | 27.84545 | 26.386-29.305 |
| January 2021 | 26.63333 | 24.351-28.916 |
| February 2021 | 29.34872 | 27.488-31.210 |
| March 2021 | 24.76522 | 23.259-26.272 |
| April 2021 | 24.46207 | 23.309-25.615 |
| May 2021 | 26.69474 | 25.025-28.364 |
| June 2021 | 32.41875 | 30.215-24.622 |
| July 2021 | 28.04167 | 24.277-31.606 |
| August 2021 | 32.125 | 30.767-33483 |
| September 2021 | 27.87273 | 26.102-29.643 |
| October 2021 | 28.64167 | 25.014-32.269 |
| November 2021 | 33.83333 | 29.994-37.673 |
| December 2021 | 30.15 | 26.715-33.585 |
